# Managing Obesity: The Need for Individualized Therapy

**DOI:** 10.3390/nu17152498

**Published:** 2025-07-30

**Authors:** Karolina Szewczyk-Golec, Iga Hołyńska-Iwan

**Affiliations:** 1 Department of Medical Biology and Biochemistry, Faculty of Medicine, Ludwik Rydygier Collegium Medicum in Bydgoszcz, Nicolaus Copernicus University in Toruń, 85-092 Bydgoszcz, Poland; 2 Department of Pathobiochemistry and Clinical Chemistry, Faculty of Pharmacy, Ludwik Rydygier Collegium Medicum in Bydgoszcz, Nicolaus Copernicus University in Toruń, 85-094 Bydgoszcz, Poland; igaholynska@cm.umk.pl

Obesity, overweight, malnutrition, and metabolic diseases have been the subject of scientific research and have attracted the attention of health organizations for years [1,2]. According to data provided by the World Health Organization (WHO), obesity and overweight are and will remain a major problem in modern society, due to the growing number of overweight and obese children and young people [2]. Obesity remains an unresolved and emotive issue. People with excess body weight are stigmatized, which further poses a challenge for those trying to lose weight. The fact that this issue remains one of the most pressing is evidenced by the development of a new definition of obesity, presented this year by an international team of experts [3]. The consequence of the new definition, related to the division of obesity into preclinical and clinical, is an individualized approach, with the definition of individual therapeutic goals tailored to the needs of the individual patient. A calorie-restricted diet is the traditional method for normalizing body weight. However, scientific research and new strategies indicate that simply restricting calorie intake is highly ineffective and, moreover, unacceptable, especially in young people and those with comorbidities. In light of these new understandings, the topic of our published Special Issue “Specialized Diet, Obesity and Associated Metabolic Diseases (Volume II)”, concerning dietary modifications to help manage obesity and its comorbidities, seems to be particularly relevant. Undoubtedly, the results of the studies described in the articles collected in our Special Issue support the need to individualize therapeutic treatment for patients with obesity.

Obesity is a condition especially difficult to treat if it is related to hormonal dysregulation of the organism. A compelling and important narrative review of nutrition, lifestyle, and cardiometabolic disorders in patients with polycystic ovary syndrome (PCOS) was presented by Herbert et al. The authors compiled data from 51 randomized controlled trials on dietary or lifestyle interventions in PCOS, and, based on this data, tried to propose efficient strategies to reduce cardiometabolic risk in PCOS patients with different body sizes. The need for lifestyle changes as a protective measure against weight gain and cardiometabolic risk in PCOS patients was emphasized. Moreover, it was concluded that less restrictive dietary approaches, focused on balanced dietary patterns tailored to the individual nutritional needs and preferences, should be developed to efficiently help PCOS subjects.

The disruption of systemic homeostasis regarding oxidants and antioxidants, along with endothelial dysfunction in patients with metabolic syndrome, was discussed by Shrebiani et al. A detailed discussion of the role, interplay, and pathophysiological network of obesity, diabetes, and hypertension in inducing oxidative stress was presented. Results on the effects of supplementation with vitamins C and E, minerals, melatonin, L-arginine, flavonoids, anthocyanins, and carotenoids on oxidative function, inflammation, and endothelial function in patients with metabolic syndrome, hypertension, obesity, dyslipidemia, and hyperinsulinemia were also discussed. The analysis conducted by the authors allows us to conclude that a holistic approach to the patient is the most effective in maintaining a healthy body weight and reducing the risk of metabolic disorders.

Research on the effectiveness of various supplements in the treatment of obesity and its complications is still the focus of interest of many research groups. The double-blind randomized control trial for a group of 60 obese men, presented by Moqaddam et al., involved supplementation with astaxanthin, a carotenoid derivative, and CrossFit training sessions. Twelve weeks of astaxanthin supplementation with training sessions were found to improve lipid profiles, reduce glucose levels, and decrease activin A, myostatin, and transforming growth factor β1 (TGF-β1) levels. The authors summarized that physical exercise, along with the use of an antioxidant factor, might be an efficient strategy to regulate adipocyte metabolism. In turn, a pilot study of beetroot juice consumption for two weeks in seven patients with type 2 diabetes, described by Tyler et al., demonstrated a reduction in systolic and diastolic blood pressure, which the authors attributed to increased NO bioavailability measured in the saliva and blood. A slight reduction in glucose levels measured by the oral glucose tolerance test was also observed. Given the promising results and the easy availability of beetroot juice, the authors emphasized the need for further studies in a larger group of patients.

Assessing the impact of the entire diet, not individual nutrients, on health indicators is also an important subject of studies on obesity. Pietrzak et al. conducted a study concerning the inflammatory potential of different diets and their impact on metabolism. The dietary Inflammatory Index (DII) was investigated in a group of 292 young adults who were regularly physically active. It was demonstrated that the lower the DII, the greater the consumption of macro- and micronutrients. Moreover, a link between higher body weight and increased DII was determined. Particularly disturbing is the fact that nearly 75% of the young people consumed foods with pro-inflammatory properties, which negatively affected their metabolism.

Research on animal models allows for a more precise understanding of the effects of specific diets or supplements. Fitzgerald et al. studied the effect of a diet containing 35 to 75% common beans on the development of metabolic dysfunction-associated steatotic liver disease (MASLD) in mice. Global metabolomic profiling of the liver and plasma enabled the identification of sphingolipids as a lipid subcategory on which bean consumption had significant effects. A reduction in lipid levels, specifically changes in sphingolipid biotransformation, in the liver of mice fed an increased content of common beans was determined. This result may have implications for nutritional support in the treatment of metabolic liver disorders. In the experiment presented by Zhao et al., mice fed a high-fat diet were supplemented for 12 weeks with a postbiotic soy protein and a prebiotic containing galactose oligosaccharides, fructose oligosaccharides, and lactitol. Body weight, blood lipid profile, and fecal lipid content were analyzed, and the gut microbiota was assessed. The authors demonstrated that postbiotic and prebiotic administration supported weight loss, the formation of lipid-rich feces, and the regulation of the gut microbiota.

Pregnancy is a unique physiological condition associated with a higher risk of developing obesity in women. Conversely, excess maternal body weight impacts fetal development and the child’s health. Therefore, research on pregnancy, obesity, and fetal health is of first importance. A properly formulated diet containing adequate nutrients is crucial for both the pregnant woman and the fetus. The article by Szczuko et al. presents the results of a study involving 78 pregnant women who were overweight or obese before and during pregnancy. High-performance liquid chromatography was used to measure blood levels of eicosanoids, including protectin DX, maresin, resolvin D1, and resolvin E1. Those eicosanoids are associated with the suppression of inflammation during gestation, which is important for the normal course of pregnancy. The overweight or obese women in the third trimester of pregnancy had significantly higher levels of protectin DX and resolvin D1, while resolvin E1 was lower. The authors attributed these results to an increased need to suppress inflammation associated with obesity during pregnancy. A lower intake of products rich in omega-3 fatty acids by obese pregnant women was also considered.

An important finding was presented by Wayland et al. regarding the vertical transmission of obesity. Their studies on the C57BL/6 mouse model, which mimics human obesity, demonstrated that maternal obesity decreased neonatal survival, increased offspring adiposity, and predisposed offspring to obesity and metabolic syndrome. Severe maternal obesity influenced the offspring microbiome and created a pro-inflammatory gestational environment, causing inflammatory changes in the offspring both in utero and adulthood. Importantly, the authors also conducted a similar analysis of a human birth cohort study of obese and nonobese mothers and their children, obtaining consistent results concerning the inflammatory state in mothers and the tendency to gain weight in children.

Prenatal alcohol exposure is linked to a wide range of developmental and behavioral disorders. The possible influence of prenatal alcohol exposure on the hormonal regulation of appetite and, therefore, eating disorders and growth and developmental delays is an interesting issue. Podgórski et al. measured the levels of hormones related to appetite regulation, including neuropeptide Y, Agouti signaling protein, alpha melanocyte-stimulating hormone, and kisspeptin, in the blood samples collected from 57 patients with fetal alcohol spectrum disorders (FASD) and 23 healthy volunteers. However, no significant differences in the levels of the hormones studied were found. Interestingly, some relationships between hormone concentrations and parameters describing the clinical status of patients with FASD were observed.

Obesity and overweight are significant problems in both developed and developing societies. Researchers around the world are addressing this global challenge by exploring it from a wide range of perspectives. The articles presented in our Special Issue reflect this diverse and multifaceted nature of research on obesity. Through these studies, researchers continue to gain new insights into this complex and still not fully understood condition, characterized by excessive adipose tissue. Better understanding allows the development of more effective preventive and treatment strategies. In particular, the studies presented in this Special Issue underscore that food quality, personalized supplementation, and regular physical activity not only support healthy body weight but also play a vital role in preventing the metabolic complications of obesity in both adults and their offspring.
